# Determining Moisture Content in Milk Powder: Challenges in the Evaluation of Performance by Proficiency Testing Using Independent Reference Values

**DOI:** 10.3390/s25051579

**Published:** 2025-03-04

**Authors:** Susan Poo, Miguel Palma, Ociel Muñoz

**Affiliations:** 1 Laboratory for Measurement Quality Assurance (LACM), Institute of Food Sciences and Technology, Faculty of Agricultural and Food Sciences, Universidad Austral de Chile, Valdivia 5090000, Chile; susan.poo@uach.cl; 2 Department of Analytical Chemistry, Center of Agri-Food and Wine Research (IVAGRO), Faculty of Science, University of Cadiz, 11510 Puerto Real, Spain; 3 Institute of Food Sciences and Technology, Faculty of Agricultural and Food Sciences, Universidad Austral de Chile, Valdivia 5090000, Chile; ocielmunoz@uach.cl

**Keywords:** proficiency testing, reference value, assigned value, small number of laboratories, milk powder, water content, moisture

## Abstract

The water content in milk powder must be controlled to ensure its stability. The analytical methods used for its determination can be verified through proficiency testing (PT). This study evaluated the reference values, their uncertainty, and the implications for nine PT rounds on moisture determination in milk powder conducted between 2017 and 2021 by a Chilean PT provider. An independent laboratory assigned the reference values, considering the analysis session as a source of uncertainty. Results showed that in 77.8% of the rounds, uncertainty did not meet the ISO 13528:2022 criterion, requiring the use of z′-score, which increased the risk of erroneous evaluations in 5.8% of cases, particularly near threshold values (z′ ≈ 2.0 or 3.0). The robust standard deviation from participants’ results exceeded 0.14 g water/100 g milk powder in 63.0% of PT items, suggesting that future evaluations should consider modifying the standard deviation for proficiency assessment. Reference values were found to be compatible with participants’ consensus values, though their use affected 19.3% of performance classifications, reinforcing the need for independent reference values in PT schemes with a small number of laboratories. These findings highlight the importance of evaluating the dispersion trends in proficiency assessment and adapting standard deviation criteria for more accurate performance evaluation.

## 1. Introduction

Water content in milk powder may affect its stability during its shelf life, as well as its solubility, wettability, and oxidative stability. To prevent milk powder from absorbing moisture from the environment, commercial products use moisture-proof packaging materials such as aluminum-laminated films, multi-layer plastic pouches, or metalized polyester films to block water vapor transmission. Additionally, they must be stored at cool temperatures, preferably below 25 °C. Given its relevance as an internationally traded product, moisture content limits have been established [1]. Such limits may vary depending on the producer, the buyer, or any regulatory specifications. Although regular control equipment has been reported to be set up for calibration at a range between 0.5 and 8.6 g water/100 g milk [2], milk powder moisture content is generally found between 2.0 and 5.0 g water/100 g milk [3,4,5]. Water can be found in food as free water, adsorbed water, or bound water, where free water is the form used by biochemical reactions and can act as a dispersing agent, as a solvent for crystalline compounds, or for microbiological growth [1].

Water content in milk powder is generally determined by gravimetric methods, which are based on the loss of mass that takes place when milk powder is heated in an oven under specific drying conditions (temperature, time, amount of sample, etc.). Some standards define this loss of mass as water content [6] but most often is defined as moisture content [7,8,9]. Some examples of these methods are described in ISO 5537:2023 [8] (87 °C with a constant flow of dry air), AOAC 927.05 [9] (100 °C in a vacuum oven), or the gravimetric method in IDF 26A 1993, where the milk powder is kept at 102 ± 2 °C [6]. The latter being the method that is most often used, even though this standard was replaced by IDF 26/ISO 5537 standard [8] in 2004 in order to improve its reproducibility. For this purpose, by applying a stream of dry air to prevent ambience moisture to affect the drying process of the gravimetric method [1]. Regarding the gravimetric method at 102 ± 2 °C, one of the main manufacturers of spray drying towers for milk powder, GEA Niro, published 2 gravimetric methods for the analysis of milk powder [7,10] based on this method. Thermogravimetric moisture analyzers and Near-Infrared Spectroscopy (NIR) are other technologies frequently used by laboratories [11].

An erroneous analytical result in the quality control of milk powder can have important economic consequences for the industry, as excessive or insufficient drying of the product may alter the sensory, nutritional, and safety properties or shelf life of the product. Given the importance of this measurement, one way to ensure its validity is through the participation of laboratories in proficiency testing (PT), being also a requirement to be met by laboratories accredited under the ISO 17025:2017 standard [12]. In each PT round, the performance of the participants is evaluated with respect to the results reported and the values assigned. In other words, besides participating in the laboratory and their performance level, the assignment of a reference value is a fundamental part of any PT data analysis. The ISO 13528:2022 standard [13] describes 5 approaches aiming to determine the reference value: formulation, usage of certified reference material or CRM, the results obtained by a laboratory using a reference method or through traceability to a CRM, the consensus value determined by expert laboratories or the consensus value of the participants’ results. In practice, a PT provider selects the most suitable approach, according to the available resources and the nature of the PT items. The participants’ consensus value has been the most widely used method for performance evaluation through PT [14,15]. Nevertheless, for PT when the number of participating laboratories is less than 30 [16], the ISO 13528:2022 standard [13], similar to the IUPAC/CITAC guide for the selection and usage of PT for a limited number of participants [16] recommends the reference value to be assigned according to a valid metrological procedure, such as formulation, CRM or the results from a laboratory. According to this guide, a PT item can be considered as in-house reference material (IHRM). Other studies have also mentioned the importance of the assignment of reference values that are metrologically traceable, i.e., obtained independently from the participants’ results, whenever this is possible [17,18,19,20,21,22].

The participants in PT must be informed of the uncertainty of the reference values [23], which, when 0.3 times lower than the standard deviation for proficiency assessment, can be considered negligible. On the other hand, when this criterion is not met, according to the ISO 13528:2022 standard [13], the PT provider should take measures, such as using the uncertainty of the performance evaluation based on the z′-score [13]. Considering the above, it is important to have in place a correct method to estimate the uncertainty, which will depend, among other things, on the approach that has been used to assign the reference value. According to Bettencourt et al. [24], the uncertainty of a chemical measurement can be estimated using approaches such as modeling, laboratory validation, or quality control, and the results from interlaboratory tests. The approach based on validation and quality control data uses the data collected over a period of time from a particular laboratory (intra-laboratory or intermediate reproducibility) [25,26]. It incorporates two aspects that may affect the calculation of the uncertainty: the metrological traceability (when using CRMs for the estimation of the bias) and the intra-laboratory precision.

Since PT items can be considered as IHRMs, the procedures used to certify reference materials could also be used for the assignment of the reference value to the PT items. Other authors [27] suggest that, for the characterization of the reference materials, the minimum possible number of determinations should be used in order to achieve a realistic and meaningful determination of the uncertainty.

The ISO 13528:2022 standard [13] describes a procedure that can be used to assign reference values independently and based on the results from a particular laboratory, for which the PT items are analyzed together with a CRM in one analysis session under repeatability conditions. The PT provider LACM^®^/Metrology Division, which organizes PT rounds that intend to ensure the validity of moisture measurements in milk powder, applies this procedure to this and other PT programs because of the small number of participants. Besides the aforementioned key aspects regarding the determination of uncertainty, other factors, such as the independence of the measurements and the analysis session, are incorporated into their design. In the present study, the effect of employing this design for the PT rounds performed between 2017 and 2021 has been investigated with regard to reference values, compliance with the ISO 13528:2022 standard [13] criterion for the uncertainty, evaluation of the performance of the participating laboratories, compatibility of the assigned values and criteria for proficiency assessment. For this purpose, the current state of development of the analytical methods has been considered. Additionally, the compatibility between the reference values assigned by calibration assays with CRM in PT rounds for laboratories and the robust consensus values obtained from the participants was determined.

## 2. Materials and Methods

### 2.1. Overview

The results from 9 PT rounds on the “Water content in milk powder” with the participation of 6 to 10 laboratories and 3 items per PT round, each one identified as 1701, 1702, 1801, 1802, 1901, 1902, 2001, 2002 and 2101 were examined. The PT rounds were conducted between the years 2017 and 2021 by the LACM^®^/Metrology Division of the Institute of Food Science and Technology (ICYTAL) at the Austral University of Chile—a PT provider that operates under the requirements of the ISO 17043:2010 standard [23].

### 2.2. Planning, Preparation, and Distribution of the PT Items

The planning, preparation, and distribution of the PT items to the participants and to the laboratory that performed the analyses to obtain the reference value and the homogeneity, were coordinated by the personnel from the Metrology Division of the LACM^®^. For the preparation of the PT items, milk powder produced by dairy industries in the South of Chile) were used. The PT items, corresponding to whole or skimmed milk powder, were distributed in 30 g portions inside trilaminate film bags, after being mixed to ensure their homogeneity. The samples were delivered to the laboratory that performed the analyses at room temperature, together with a CRM of milk powder (with similar fat content as the PT items) to perform the calibration or traceability assay required for the assignment of reference values. According to the reports from the PT provider, the PT items complied with the homogeneity and stability requirements. The CRM batches (MP-0210, MP-0216, and MP-0212) were purchased from a German reference material producer (muva Kempten, Kempten, Germany).

### 2.3. Assignment of Reference Value

#### 2.3.1. Experimental Design

The design used to assign the reference values consisted of a calibration assay through the analysis of the PT items together with a CRM. This design features a modification with respect to the one described in the ISO 13528:2022 standard [13] with regard to the number of samples and the effect attributable to the analysis session [25,27].

As an integral part of the experimental design, each PT round comprised 4 sessions, where a sample from each PT item and 2 CRM replicas were analyzed per session.

#### 2.3.2. Calibration Assay to Assign the Reference Value

The PT item samples and the CRM were analyzed through the gravimetric method at 102 °C, using a procedure based on the IDF/IDF 26A:1993 standard [6]. These analyses were performed by the LACM^®^/Analytical Division laboratory of the Universidad Austral de Chile, according to the requirements of the NCh-ISO 17025:2017 standard [12].

For the calculation of the reference value, the terms of the equation described in the ISO 13528:2022 standard [13] were rearranged, taking as reference the work published by Poo et al. [28], and Equation (1) was obtained:

(1) $$\hat{x}={\bar{x}}_{PTI}-\left({\bar{x}}_{CRM}-c_{CRM}\right)$$

where:

$\hat{x}$ reference value (assigned value) for the PT item,
${\bar{x}}_{PTI}$ the average result corresponding to the *n* replicates of the PT item
${\bar{x}}_{CRM}$ the average result corresponding to the *n* replicates of the *CRM* analysis, and
$c_{CRM}$ the certified reference value of the *CRM*,

The compatibility of the assigned value with the results from the participants (robust consensus mean) was also evaluated, by calculating the differences between both values and by comparing them against the expected difference according to the precision of the analytical method [16,28]. The effect of using a consensus value for the evaluation of the laboratory performance (z-score) in comparison to using the reference value obtained through a calibration assay was also studied [22,29].

#### 2.3.3. Standard Uncertainty of Reference Values

In accordance with the criteria established by the ISO 13528:2022 standard [13], the standard uncertainty of the assigned value $u_{\hat{x}}$ can be neglected when the criterion $u_{\hat{x}}<0.3\hat{\sigma }$ is met. In this case $\hat{\sigma }$ is the standard deviation for the proficiency assessment. It is used to evaluate the performance of the participants based on z-score. When the above-mentioned criterion is not met, one of the options consists in incorporating the uncertainty into the evaluation of the performance using z′score [13]. Therefore, for each PT round, a calculation of the standard uncertainty of the assigned values was performed to determine compliance with such criterion.

Keeping in mind the calibration assay on a CRM as well as a paper recently published on proficiency testing for fat and crude protein in milk [28], the standard uncertainty of the assigned values was determined by means of the law for the propagation of uncertainties [30] using Equation (2):

(2) $$u_{\hat{x}}=\sqrt{\left(u_{{\bar{x}}_{PTI}}^{2}+u_{{\bar{x}}_{CRM}}^{2}+u_{CRM}^{2}\right)}$$

where: $u_{{\bar{x}}_{PTI}}$ is the standard uncertainty of the assay on the PT item, $u_{{\bar{x}}_{CRM}}$ is the standard uncertainty of the assay on the CRM, and $u_{CRM}$ is the standard uncertainty of the CRM, which are obtained from Equations (3)–(5):

(3) $$u_{{\bar{x}}_{PTI}}=\sqrt{\left(\frac{s_{session}^{2}}{n}+\frac{s_{r}^{2}}{nq}\right)}$$

where: $s_{session}$ is the standard deviation between sessions (obtained from the pooled standard deviation of the results of the 3 PT items in each round), *n* is the number of sessions (4 in this case), *q* is the number of replicas, and $s_{r}$ is the standard deviation of the repeatability (which is obtained through an ANOVA considering the session as a random factor).

(4) $$u_{{\bar{x}}_{CRM}}=\sqrt{\left(\frac{s_{session}^{2}}{n}+\frac{s_{r}^{2}}{nq}\right)}$$

where $s_{session}$ and $s_{r}$ are obtained from an ANOVA (“session” is considered a random factor).

(5) $$u_{CRM}=\frac{U_{CRM}}{k}$$

where $U_{CRM}$ corresponds to the expanded uncertainty ($U_{CRM}$) and k is the coverage factor, both of which are stated by the manufacturer of the CRM on its certificate.

#### 2.3.4. Compliance with the Requirements for Uncertainty of the Reference Values and Implications for the Performance Evaluation

Besides the compliance with the ISO 13528:2022 standard criterion [13] with respect to the uncertainty of the assigned values, the contribution from the various uncertainty sources was assessed and compared against the values indicated by the provider on their design documentation [31].

For the calculations and the statistical analyses, MS Excel 2016 (Microsoft, Redmong, WA, USA) spreadsheets, the R Studio 4.4.1 software package by RobStatTM (Boston, MA, USA), and the SPC statistical software for Excel 5.0.2.1 (BPI Consulting LLC, Oklahoma City, OK, USA) were employed.

### 2.4. Criteria for the Proficiency Testing of the Participants

In order to determine if the criteria for the performance evaluation of the participants were appropriate, the standard deviation values for the proficiency assessment $\hat{\sigma }$ established by the PT provider [31] based on the precision indexes of the different analytical methods used by the participants [13,32], were reviewed according to the state of the art of the analytical methods and a comparison with the consensus value of the standard deviation (robust standard deviation of the participants’ results) obtained through the *Qn* estimator [13].

#### 2.4.1. Criteria for the Gravimetric Method at 102 °C

The standard deviation of the proficiency test was determined through Equation (6) [13]:

(6) $$\hat{\sigma }=\sqrt{\left(\sigma_{R}^{2}-\sigma_{r}^{2}+\sigma_{r}^{2}/m\right)}$$

where $\sigma_{R}$ is the standard deviation of the reproducibility, $\sigma_{r}\;$ is the standard deviation of the repeatability and *m* is the number of replicas per participant. For the calculation, the standard deviation of the repeatability and of the reproducibility in a collaborative study mentioned in IDF 26A:1993 was considered [6], where 0.071 g of water/100 g of milk powder and 0.143 g of water/100 g of milk powder were considered respectively. The result obtained from Equation (6), rounded to the nearest hundredth was 0.14 g of water/100 g of milk powder.

#### 2.4.2. Criteria for the Near-Infrared Spectroscopy (NIR) Method

In order to determine the standard deviation for the proficiency assessment of the NIR method, the PT provider considered the criteria established in ISO 21543:2020(E)/IDF 201:2020(E) [11] in relation to the precision error. This error is derived from the calibration, and its magnitude is equal to the SEP (standard error of prediction):

(7) $$SEP=\sqrt{\sum \frac{1}{N-1}{\left(y_{i-NIR}-y_{i-ref}-B\right)}^{2}}$$

where $\left(y_{i-NIR}-y_{i-ref}\right)$ is the difference between the result obtained by the NIR method ($y_{i-NIR}$) and the reference method ($y_{i-ref}$) for the sample *i*, *N* is the total number of samples and *B* is the bias, represented by the expression

(8) $$B=\frac{1}{N}\sum \left(y_{i-NIR}-y_{i-ref}\right)$$

The previously mentioned standard [11] provides some examples of SEP values for moisture in milk powder that ranges between 0.08 and 0.27 g of water/100 g of milk powder. On the other hand, the manufacturer of the NIR equipment used by the Chilean laboratories (Foss), indicates a value of 0.15 g of water/100 g of milk powder [2]. Based on this information, the standard deviation for proficiency assessment was assigned in common with that of the gravimetric method, following the “fitness for purpose” criterion, which was $\hat{\sigma }$ = 0.14 g of water/100 g of milk powder.

#### 2.4.3. Thermogravimetric Moisture Analyzer

The information provided by the manufacturers of thermogravimetric moisture analyzers has been considered, since there are no standards to establish their accuracy. According to one report [33], the accuracy range that can be achieved with the different variations of the gravimetric method would be the same regardless of the gravimetric method used (oven, infrared, halogen, microwave) and would go from 0.1 to 0.5% moisture. The target calculated for the other analytical methods corresponds to an accuracy of 0.28 g of water/100 g of milk powder (i.e., 2 × 0.14 g of water/100 g of milk powder), which is slightly higher than half this range. In other words, it is possible to apply this target to a thermogravimetric moisture analyzer if the appropriate conditions are selected (time, temperature, drying process completion criterion), for which the fitness for purpose criterion was applied, where $\hat{\sigma }$ = 0.14 g of water/100 g of milk powder.

## 3. Results and Discussion

### 3.1. Reference Values Assigned to the PT Items

The reference values assigned to the PT items ranged from 2.68 to 3.88 g of water/100 g of milk powder (Appendix A). An average bias of −0.049 g of water/100 g of milk powder was determined based on the results obtained from the CRM assays in all the rounds (Appendix A). Based on the analyses of the CRM in previous PT rounds [31], it was higher (in absolute terms) than the bias estimated by the PT provider (−0.025 g of water/100 g milk powder). Although the Student’s *t*-test statistic indicates at 95% confidence that the bias is statistically different from zero (*p*-value 0.018), its magnitude would not be significant from a metrological point of view, as it is lower than the repeatability of the method (0.20 g of water/100 g milk powder) [6].

In order to determine their compatibility, Section 3.4 includes the comparison between the reference values and the robust consensus mean of the participants’ results.

### 3.2. Standard Uncertainty of the Assigned Values

Table 1 includes the standard uncertainty of the reference values obtained through Equation (2) according to the different sources used for its calculation.

It can be seen from Table 1 that in 2 of the 9 rounds (H LP 1702 and H LP 1802), i.e., 22.2% of the rounds, the standard uncertainty of the reference values meets the criterion mentioned in the ISO 13528:2022 standard [13], namely to be less than $0.3\hat{\sigma }$ (0.042 g of water/100 g of milk powder), with H LP 2101 round being an edge case. The standard deviation of the proficiency test ($\hat{\sigma }$) was 0.14 g of water/100 g of milk powder for all the analytical methods. According to these calculations, in the rounds, it would not be necessary to incorporate the uncertainty into the evaluation of the performance of the participants, and a z-score could be used. In the rounds where the criteria of the ISO 13528:2022 standard [13] are not fulfilled, the performance of the participants can be evaluated through the calculation of z′-score, which would have the following implications: The ratio between the uncertainty and the standard deviation for the proficiency assessment (Table 1) would be less than or equal to 0.40 for most of the rounds in which z′-score is used (6 out of 7). By incorporating the uncertainty into the evaluation of performance, the z′-score values would correspond to 0.93 to 0.96 of the z-score value, which is calculated by applying the following equation [13]:

(9) $$\frac{z^{\prime }-score}{z-score}=\frac{\hat{\sigma }}{\sqrt{\left({\hat{\sigma }}^{2}+u_{\hat{x}}^{2}\right)}}$$

For example, when $u_{\hat{x}}$ = 0.4$\hat{\sigma }$, a z-score = 3.00 would be equivalent to a z′-score = 2.79 (0.93 factor); whereas for rounds in which the uncertainty is equal to 0.3$\hat{\sigma }$, a z′-score equal to 2.88 (0.96 factor) would be obtained. For the only PT round in which the ratio $u_{x}/\hat{\sigma }$ exceeds 0.40 (H LP 1701), the factor is 0.91. Considering this, the risk of misevaluating the participants would occur in those edge cases, or those near to |z| = 2 or |z| = 3 (considering that when 2 < |z or z′| ≤ 3 the participant’s performance is questionable and when |z or z′| ≥ 3 their performance is unsatisfactory). It would affect 3.9% of the total number of participants in the evaluated PT rounds, for whom performance would change from questionable to satisfactory when using z′-score and also 1.9% of the participants, whose performance would change from unsatisfactory to questionable. Therefore, it would make a total of 5.8% participations affected (considering 3 PT items being analyzed in each of the 9 rounds).

On the other hand, the components that contribute most to the estimation of the uncertainty of the reference values (Table 1) are, in the first place, those of the PT item test ($u_{{\bar{x}}_{PTI}}$). The uncertainty of the CRM-certified value $(u_{CRM}$) is the second most significant contributor to the uncertainty of the assigned value, which in 100% of the rounds exceeded by more than 2 fold the value estimated by the design (0.015 g of water/100 g of milk powder) [31]. With respect to the uncertainty obtained from the CRM assay ($u_{{\bar{x}}_{CRM}}$) a value lower than or equal to the design estimate (0.033 g of water/100 g of milk powder) [31] was obtained in 100% of the PT rounds. The contributions from the different sources are similar in order of importance to those found in another PT scheme for which a similar design had been applied [28].

### 3.3. Criteria for the Evaluation of the Participants’ Performance

The results obtained from the review of the analytical methods used by the participants are presented below in order to determine whether the precision indices considered for the calculation of the standard deviation for the proficiency assessment $\hat{\sigma }$ are currently valid.

#### 3.3.1. Gravimetric Method (Drying Oven at 102 °C)

For this method, $\hat{\sigma }$ was established based on the precision data required by the IDF 26A:1993 standard [6]. No later update of the standard has been published for the method using 102 °C, as it was replaced by the standard IDF 26 (ISO 5537) [8]. In this case, the drying temperature is 87 °C and a specific equipment is used (temperature-controlled drying oven with forced ventilation and the specific characteristics described in the standard IDF 26 [8]), according to which, a constant flow of dry air is applied. Participating laboratories do not currently use the reference method so that assigning the reference value according to the method using 102 °C is considered appropriate. Other methods that use the traditional drying oven method at 102 °C have been reviewed and the following aspects were found: The GEA Niro analytical methods [7,10] would present a reproducibility of ±0.1%, which would be more demanding and would not be compatible with the other methods. On the other hand, the PT provider suggests that the moisture content in a milk powder sample, analyzed for quality purposes and in compliance with specifications, is between 2.2 and 5.0 g of water/100 g of milk powder, i.e., a 2.8 g of water/100 g of milk powder range. The expanded uncertainty of the analytical method should not be greater than 1/8 that range, in accordance with the ASME B89.7.3.1-2001 standard [34], namely 0.35 g of water/100 g of milk powder, which would be a standard uncertainty equal to 0.35/2 = 0.18 g of water/100 g of milk powder (for a k = 2 coverage factor). Therefore the standard deviation of the proficiency test 0.14 g of water/100 g of milk powder, would be below the maximum uncertainty (0.18 g of water/100 g of milk powder). It means it would be suitable to determine if a method fulfills the fitness-for-purpose criterion. Nevertheless, when the aforementioned range between 2.0 and 5.0 g of water/100 g of milk powder [3,4,5] is considered and this criterion is applied, the expanded uncertainty of the analytical method would be 0.38 g of water/100 g of milk powder. The standard uncertainty would be 0.19 g of water/100 g of milk powder, which means, the standard deviation for the proficiency assessment could be established between 0.14 and 0.19 g of water/100 g of milk powder.

#### 3.3.2. Near-Infrared Spectroscopy (NIR) Method

The calculation of the standard deviation for the proficiency assessment for the NIR method was based on the standard ISO 21543:2020 (E)/IDF 201:2020 (E) [11] without any modification with regard to the standard error of prediction (SEP) values presented as an example. Therefore, according to this criterion, the value of $\hat{\sigma }$ = 0.14 g of water/100 g of milk powder could remain invariable for the subsequent PT rounds of the participants using the NIR method.

#### 3.3.3. Thermogravimetric Analyzer

The standard deviation for the proficiency assessment in this method is based on the specifications provided by the suppliers of the thermogravimetric moisture analyzers. Ohaus [35] and Mettler-Toledo [36] specifications indicate standard deviation values for repeatability between 0.015 and 0.15 g of water/100 g of milk powder that vary with the number of samples to be analyzed, where most are compatible with the repeatability of the drying oven method at 102 °C [6] (0.20 g of water/100 g of milk powder), which has been considered for the calculation of the proficiency test standard deviation. Therefore, after this review and in view of the data presented in Section 2.4.3, the fitness for purpose criterion should remain in place.

#### 3.3.4. Standard Deviation of the Proficiency Assessment with Respect to the Standard Deviation of the Participants’ Results

**Figure 1:**
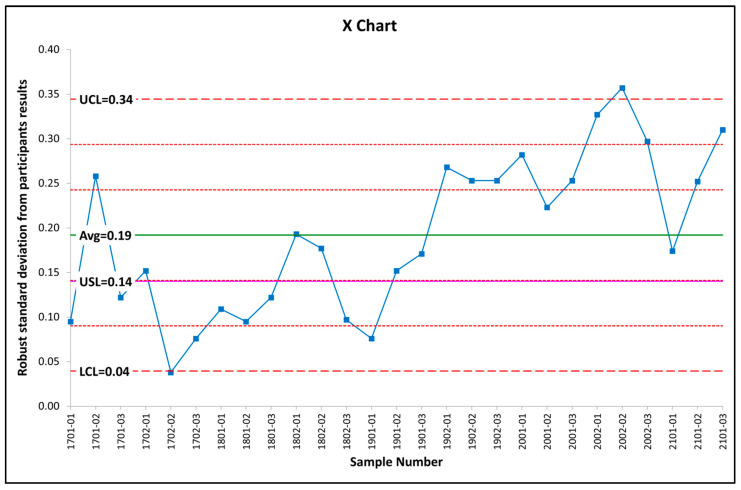
Robust standard deviation of the participants in the PT rounds held between 2017 and 2021 compared to the standard deviation for the proficiency assessment. UCL = Upper Control Limit; Avg = mean; USL = Upper Specification Limit (standard deviation of the proficiency test); LCL = Lower Control Limit.

In order to determine if the laboratories achieve standard deviation results that are lower than or equal to the value established for the standard deviation of the proficiency test (0.14 g of water/100 g of milk powder), the robust standard deviation of the participants’ results was determined using a *Qn* robust scale estimator [13]. Since this criterion is common to all the methods, all the participants were included for determination, regardless of the analytical method used. The results are presented in Figure 1.

In general, PT schemes that are open to the participation of different laboratories. Errors would be expected to decrease over time as the experience of the participants increases and the consensus among them improves. Therefore it would lead to a decreasing dispersion of the results [37], however, for this particular PT scheme, the number and the identity of the laboratories can change from one round to the next one, so a reduction of the dispersion is not necessarily to be expected. It can be seen from Figure 1 that, only for 37.0% of the PT items, the laboratories could achieve a robust standard deviation below the $\hat{\sigma }$ value (Upper Specification Limit, USL), with values increasing from PT 1902 onwards. On the other hand, all the analytical methods have been accounted for in the results, which could also mean that the target is not appropriate for all of these methods. It is suggested to continue evaluating the standard deviation of the participants’ results through subsequent rounds, as it might be necessary to modify this criterion and consider, for example, the average value of 0.19 g of water/100 g of milk powder, which coincides with the maximum uncertainty proposed in Section 3.3.1 and would allow the compliance with the criterion for uncertainty established by the ISO 13528 standard [13] in 8 of the 9 rounds (Table 1). On the other hand, with the current $\hat{\sigma }$ value (0.14 g of water/100 g of milk powder), the need to use z′-score is equivalent to increasing the standard deviation for the proficiency assessment from 0.14 to values between 0.15 and 0.21 g of water/100 g of milk powder. Finally, the SEP value specified in the standard for the NIR method is 0.15 g of water/100 g of milk powder [11], so an increase in the standard deviation for the proficiency would be taking into account the precision of this method.

### 3.4. Comparison Between Consensus and Reference Values

**Figure 2:**
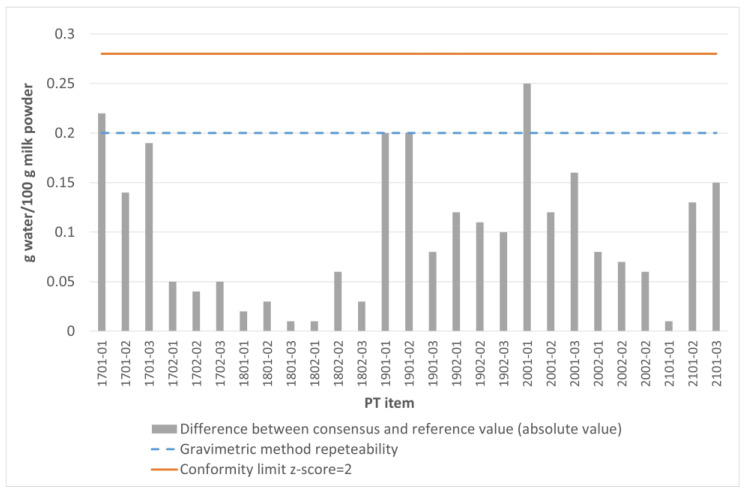
Difference between robust consensus average of the participants’ results and reference values assigned by calibration essay with CRM.

The reference values were compared against the robust consensus average of the participants’ results (Figure 2). The absolute difference between consensus and reference value is shown. Tukey’s Biweight robust estimator was used to calculate the consensus average [38].

**Figure 3:**
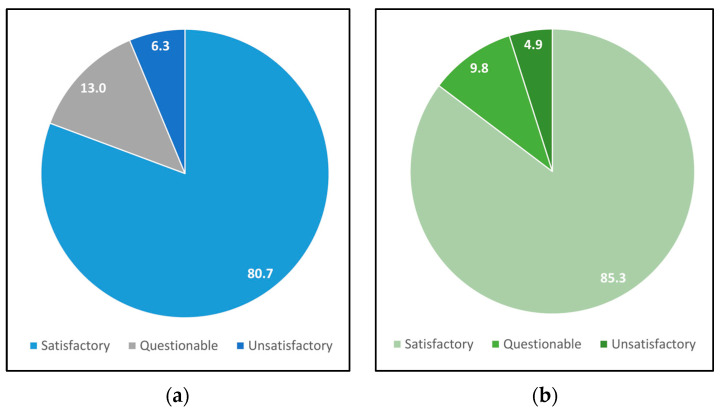
Laboratory performance measured by z-score when using reference values (**a**) and when using consensus values (**b**).

The average difference between the robust consensus average and the reference value is −0.07 g of water/100 g of milk powder, ranging from −0.25 to 0.15 g of water/100 g of milk powder. The magnitude of the average difference is lower than the repeatability of the method [6] which is equal to 0.20 g of water/100 g of milk powder. It is also lower than the maximum difference accepted for a laboratory to comply with a z-score = 2, which is equivalent to 0.28 g of water/100 g of milk powder for this PT. The difference between both values would be comparable to the combined uncertainty of both values [13,28], when the uncertainty is calculated based on the precision indices and on the values given in Section 2.4.1 a maximum difference of 0.40 g of water/100 g of milk powder is to be expected, which is met in all the cases. On the other hand, the percentage of laboratories whose performance would be rated as satisfactory, questionable, or unsatisfactory was determined on the basis of the z-score calculated using the reference values obtained through the calibration assay and also through the consensus values [22]. The results are presented in Figure 3.

From Figure 3, the total percentage of laboratories whose performance was satisfactory would increase from 80.7% to 85.3% when the consensus value was used to calculate the z-score. The percentage of laboratories whose performance would be questionable would decrease from 13.0% to 9.8% and the percentage of laboratories whose performance would be unsatisfactory would decrease from 6.3% to 4.9%. Moreover, the percentage of laboratories whose classification would change is 19.3% altogether, when considering the total number of PT rounds and items evaluated. Additionally, the effect of the assigned value on the z-score is statistically significant (*p*-value 0.007), which corroborates that when the consensus values were used as reference values, these could be biased, especially if the number of participants was small and new participants were incorporated [37].

## 4. Conclusions

Based on the procedures applied in the present investigation to estimate the uncertainty of the assigned values, the criterion established by the ISO 13528:2022 standard [13] for uncertainty is met in 2 of the 9 PT rounds that have been investigated. For the remaining ones, it would be necessary to use z′-score, which slightly increases the risk of an erroneous performance evaluation, especially in edge cases (z′-score close to 2.0 or 3.0), which corresponds to 5.8% of the total number of participations.

Regarding the standard deviation for the proficiency assessment, it has been observed that the precision indexes of the analytical methods have not changed, so that the fitness for purpose criterion based on the gravimetric method at 102 °C could be maintained. However, the robust standard deviation of the laboratories exceeded 0.14 g of water/100 g of milk powder value for 63.0% of the PT items tested, and increased particularly from around 1902 onwards, with an average value of 0.19 g of water/100 g of milk powder. A further evaluation of the dispersion of the laboratories is recommended, since if this dispersion remains, the value of the robust standard deviation should probably be modified, which would also increase the proportion of rounds evaluated through z-score (instead of z′-score) from 22.2 to 88.8%. Thus, would reduce the risk of erroneous performance evaluations. On the other hand, the SEP value specified in the standard for the NIR method is 0.15 g of water/100 g of milk powder, so a higher standard deviation value of the proficiency tests would allow a more appropriate performance evaluation of the participants who use the NIR method.

The reference values that were assigned by calibration assays with CRM in PT rounds for laboratories analyzing the water content in milk powder proved to be compatible with the value that could be obtained based on the participants’ results (robust consensus value). When the use of either reference values or robust average consensus is compared, significant differences can be observed with respect to compliant, questionable, or unsatisfactory z-score evaluations. It would affect 19.3% of the assessments and corroborates the significance of using values that are independent of the participants in those PTs where a reduced number of laboratories are involved.

It could, therefore, be concluded that the use of a design for the assignment of a reference value would be generally suitable for proficiency testing with a small number of participating laboratories, as long as the above-mentioned considerations are taken into account.

## Figures and Tables

**Table 1:** Combined standard uncertainty of the reference values (and its components expressed as g of water/100 g of milk powder) of the PT rounds on water content in milk powder conducted between 2017 and 2021 and uncertainty criteria.

PT ID Code	Standard Uncertainty of CRM Certificate $u_{CRM}$	Standard Uncertainty of the CRM Analysis $u_{{\bar{x}}_{\mathrm{CRM}}}$	Standard Uncertainty of the PT Item Analysis $u_{{\bar{x}}_{\mathrm{PTI}}}$	Combined Standard Uncertainty of the Reference Value $u_{\hat{x}}$	ISO 13528 Uncertainty Criteria for the Reference Value $u_{\hat{x}}<0.3\hat{\sigma }$	Ratio $\frac{u_{\hat{x}}}{\hat{\sigma }}$
H LP 1701	0.020	0.032	0.052	0.064	0.042	0.46
H LP 1702	0.020	0.010	0.024	0.032	0.042	0.23
H LP 1801	0.020	0.033	0.037	0.053	0.042	0.38
H LP 1802	0.020	0.018	0.020	0.033	0.042	0.24
H LP 1901	0.040	0.015	0.015	0.045	0.042	0.32
H LP 1902	0.040	0.014	0.036	0.055	0.042	0.40
H LP 2001	0.040	0.024	0.024	0.052	0.042	0.37
H LP 2002	0.040	0.026	0.026	0.054	0.042	0.39
H LP 2101	0.035	0.009	0.023	0.043	0.042	0.30

## Data Availability

Restrictions apply to the availability of these data. Data was obtained from the Metrology Division of the Laboratory for Measurement Quality Assurance LACM^®^ and are available on request to the corresponding author with the permission of LACM^®^. The data are not publicly available due to confidentiality agreements with the PT participants.

## Appendix A

**Table A1:** The bias of the assays for water content in milk powder on the certified reference materials (CRM) in each PT round performed between 2017 and 2021.

PT Code	Average ${\overset{=}{x}}_{\mathrm{CRM}}$ (g of Water/100 g of Milk Powder)	CRM Assigned Value $c_{\mathrm{CRM}}$ (g of Water/100 g of Milk Powder)	Bias $\left({\overset{=}{x}}_{\mathrm{CRM}}-c_{\mathrm{CRM}}\right)$ (g of Water/100 g of Milk Powder)
H LP 1701	3.92	4.00	−0.08
H LP 1702	3.92	4.00	−0.08
H LP 1801	3.97	4.00	−0.03
H LP 1802	3.96	4.00	−0.04
H LP 1901	3.37	3.49	−0.12
H LP 1902	3.43	3.49	−0.06
H LP 2001	3.44	3.49	−0.05
H LP 2002	3.45	3.49	−0.04
H LP 2101	3.27	3.21	0.06
